# Indoor radon survey in a university campus of Nigeria

**DOI:** 10.4103/0971-6203.71760

**Published:** 2010

**Authors:** R. I. Obed, H. T. Lateef, A. K. Ademola

**Affiliations:** Department of Physics, University of Ibadan, Nigeria; 1 Department of Physical Sciences, Bells University of Technology, Ota, Ogun State, Nigeria

**Keywords:** CR-39, radiation protection, radon concentration, workplaces monitoring

## Abstract

CR-39 tracketch detectors were used for the measurement of ^222^Rn concentration in 24 offices in Nigeria’s oldest university campus in order to estimate the effective dose to the occupants from ^222^Rn and its progeny. The dosimetric measurements were made over a period of 3 months. Questionnaires were distributed and analyzed. The radon concentration ranged from 157 to 495 Bq/m^3^, with an arithmetic mean and standard deviation of 293.3 and 79.6 Bq/ m^3^, respectively. The effective dose to the workers was estimated and this varied from 0.99 to 3.12 mSv/ y, with a mean of 1.85 mSv/y. The radon concentrations were found to be within the reference levels of ICRP.

## Introduction

Radon and its short-lived decay products in dwellings represent the main source of public exposure to natural radiation, contributing nearly 50% of the global effective dose to the population.[1] The naturally radioactive noble gas ^222^Rn is present in the air outdoors and in all buildings, including in workplaces. It is thus an inescapable source of radiation exposure both at home and at work.[2] Soil is considered to be the main source of indoor radon concentration, although building materials (especially quartz, cement, etc.) can make a significant contribution to the level of natural radioactivity in closed spaces such as stores and badly ventilated dwellings. Radon gas can diffuse easily out of the soil into the air or into houses. It can get trapped in poorly ventilated dwellings and its concentration can build up to high levels. Inhalation of alpha particles from ^222^Rn and associated ionizing decay products are known to cause morbidity in humans.[3] Recent researches on natural radiation exposure have recognized ^222^Rn and its progeny inside houses as a worldwide problem and a significant risk factor for lung cancer[2] It has been estimated that an increase in radon concentration of 100 Bq/m^3^is associated with approximately a 16% increased chance of developing lung cancer.[4] Literature reports of the absorbed doses to lung varies from 5 to 71 nGy/ (Bq h/ m^3^) and is summarized in Table 26 of the UNSCEAR 2000[5] report. The central value, which is estimated to be 9 nGy/ (Bq h/m^3^), was used for calculating the range of the internal effective dose equivalent rate for the population. For calculating the effective dose equivalent rate, the radon concentration measured in these offices was multiplied with the equilibrium factor (F) of 0.4 to convert it to the equilibrium equivalent concentration of radon.

It is of great importance to assess the exposure to ^222^Rn and its progeny in dwellings, especially houses, offices, and schools, for the purposes of quality control. In this context, our group is actively involved in carrying out measurements of indoor radon levels.

During the past two decades, many investigators in different countries have studied indoor radon levels.[6–8] There has been an increase in the number of studies being carried out in normal workplaces such as schools and offices.[9,10] To the best of our knowledge, no systematic study has been performed in Nigeria that has investigated radon levels in offices using CR-39 nuclear track detectors; however, some data has been published on radon levels.[11] The aim of this work was to determine the radon concentrations in 24 offices in Nigeria’s oldest university campus using, for the first time, CR-39 track etch detectors. With this study we also aimto create interest and increase public awareness about the radon hazard in the community.

## Materials and Methods

### The study location and measurement sites

**Figure 1:**
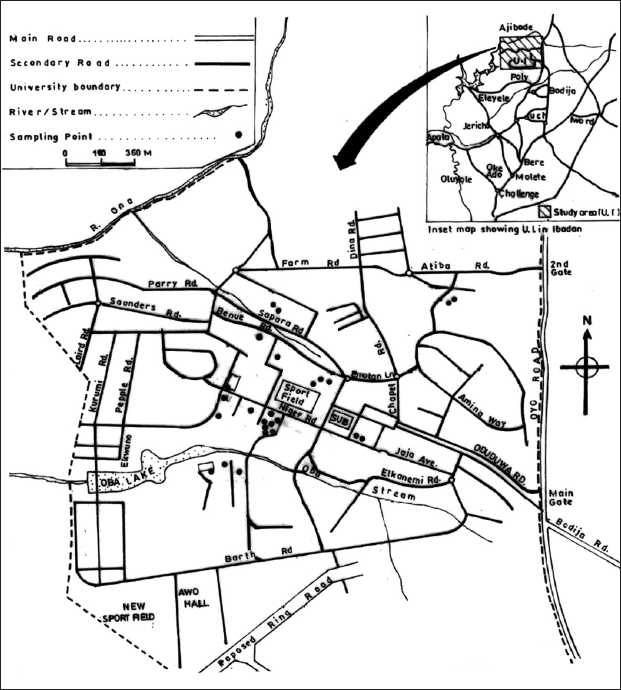
Map of University of Ibadan showing sampling points

The University of Ibadan (UI) is the oldest university in Nigeria. It is located in Ibadan, one of the three largest cities in Africa. The university has been functioning from its current site since 17^th^ November 1948 and can boast of a very well-laid out and serene campus environment. The map of the UI, including the sampling points, is shown in Figure 1.

Our survey of radon concentration in the campus to estimate the effective dose to the public from ^222^Rn and its progeny was limited to 24 offices chosen randomly from among 15 departments because of the limited number of detectors that we had and also due to the difficulty in placing the detectors in various offices. ^222^Rn concentrations in dwellings depend on meteorological and geological conditions, lifestyle, construction materials, and ventilation.[12] A questionnaire was developed and filled in by our group for every office surveyed, the information being provided mainly by its occupant(s). Apart from office identification data (occupant’s name, address, numbers of years spent in the office, etc.), the following information were obtained: floor level, wall material type, typical covering materials on internal building surfaces, year of building construction, number of smokers and number of cigarettes they smoked per day, source of tap water, floor type, presence of air conditioners, ventilation, and the underlying soil type.

### Indoor radon survey using cr-39 track etch detectors

The technique used in this survey is based on passive nuclear track detectors (NTDs), which is commercially marketed as CR-39, poly-allyl diglycol carbonate (PADC) Tastrak. These detectors are manufactured by TASL (Track Analysis Systems Ltd, Bristol, UK). They are square in shape, 1.0 × 1.0 cm in size, and have a density of 1.30 g/cm^3^. The CR-39 is a small piece of plastic that is sensitive to tracks of highly ionizing particles such as alpha particles. Radon concentration measurements were performed using CR-39 detectors enclosed in small cylindrical (5-cm height, 3-cm diameter) diffusion chambers. Detectors were etched in 6.2 M NaOH at 90°C for 2 hours, washed in clean water for 10 minutes, and then air-dried. The tracks on the CR-39 were counted with an automatic setup consisting of an optical microscope connected to a charge coupled display camera controlled by a personal computer. The calibration factor for converting track density to ^222^Rn concentration was obtained in a calibration experiment performed at the Radioactivity Laboratory of the Department of Physics, University of Trieste, Italy. These procedures were conducted in accordance with US Environmental Protection Authority Quality Assurance/Quality Control documents.[13]

The measurements of ^222^Rn concentration were performed for a period of 3 months from October 2007 to January 2008. The dosimeter at each site was hung from the ceiling at approximately 2 meters from the floor (breathing zone). After the 3-month period of exposure, 24 samples were retrieved; one detector was mishandled by an occupant in one of the offices because of fear of some diabolic intent and due to lack of awareness of the health hazard posed by indoor radon

## Results and Discussion

### Mean ^222^Rn concentration in the offices

**Figure 2:**
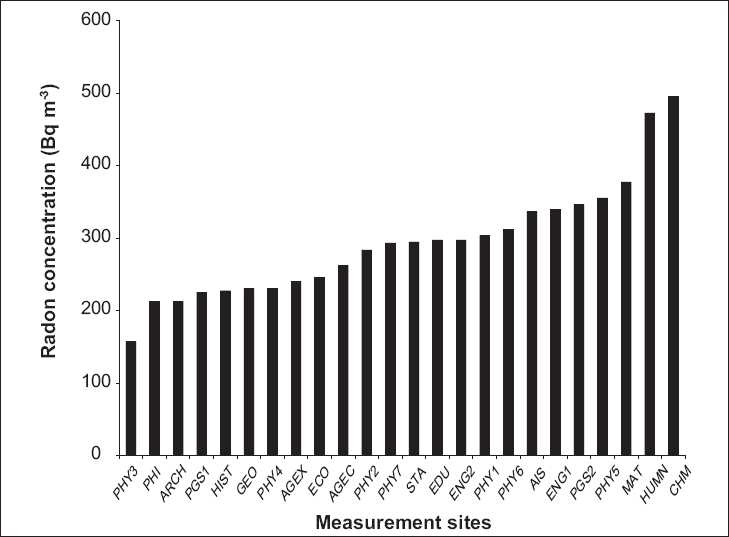
Variation of radon concentration in the offices

The track densities found on the analyzed NTDs were converted into radon concentrations (Bq/ m^3^) using the calibration factor of 1610 Bq/ m^3^(tracks/mm^2^)/day for the utilized radon dosimeter. The results for 24 sites were obtained. The variation of radon concentration in the 24 offices surveyed is shown in Figure 2. The geometric mean was 283.6 Bq/m^3^. The arithmetic mean, standard deviation, and range of ^222^Rn concentration in the offices were 293.3, 79.6, and 157–495 Bq/m^3^, respectively [Table 1]. The values are below the upper value of the ICRP reference levels of 1500 Bq/m^3^.[2]

**Table 1:** Distribution of radon concentration and annual effective dose in the offices surveyed

*Sites*	*No. of occupants*	*Years spent in office (years)*	*Radon concentration (Bq/ m^3^)*	*Estimated annual effective dose (mSv /y)*	*Accumulated dose (mSv)*
Economics (ECO)	2	3	245	1.55	4.64
Archeology (ARCH)	1	7	212	1.34	9.36
Physics (PHY1)	1	3	303	1.91	5.73
Physics (PHY2)	1	6	283	1.78	10.71
Mathematics (MAT)	1	7	377	2.38	16.64
Physics (PHY3)		2	157	0.99	1.98
Physics (PHY4)	1	15	230	1.45	21.76
History (HIST)	1	2	227	1.43	2.86
Agric. extension (AGEX)	1	5	240	1.51	7.57
English (ENG1)	1	6	339	2.14	12.83
Postgraduate school (PGS1)	1	3	225	1.42	4.26
Physics (PHY5)	1	6	355	2.24	13.43
Human nutrition (HUMN)		2	472	2.98	5.95
Arabic and islamic (AIS)	1	1	336	2.12	2.12
Geology (GEO)	1	6	230	1.45	8.70
Agric. economics (AGEC)	1	5	262	1.65	8.26
Statistics (STA)	1	2	294	1.85	3.71
English (ENG2)	1	5	297	1.87	9.37
Chemistry (CHM)	1	7	495	3.12	21.85
Physics (PHY6)	1	10	311	1.96	19.62
Postgraduate school (PGS2)	1	5	346	2.18	10.91
Education (EDU)	2	8	297	1.87	14.99
Philosophy (PHI)	2	1	212	1.34	1.34
Physics (PHY7)	1	9.5	293	1.85	17.56

### Dose estimation

The annual mean effective dose to the occupants of these offices due to exposure to radon and its progeny was estimated using the following (UNSCEAR 2000)[5]:

(1) $$\mathrm{Annual}\;\mathrm{effective}\;\mathrm{dose}\;(E)\;=\;A_{\mathrm{Rn}}.F.O.T.\mathrm{DCF}$$

where, A_Rn_is the ^222^Rn concentration (Bq/m^3^), F is the equilibrium equivalent concentration (ECC) factor for indoor exposure (0.4), O is the occupancy factor, T is the number of hours in a year (8760 h/y), and DCF is the dose conversion factor [9 × 10^6^mSv/ (Bq h/m^3^)]. All these factors, except A _Rn_, are given in the UNSCEAR report.[5] According to the mean working hours of the occupants (8 hours a day), the occupancy factor for the indoor radon exposure was estimated to be 0.2, which is the same as the occupancy factor for the outdoor exposure to terrestrial radiation in the UNSCEAR report. The values of annual effective doses thus calculated for radon inhalation by the inhabitants were found to vary in the range 0.99–3.12 mSv/ y, with a mean of 1.85 mSv/y^1^ [Table 1]. These values are higher than the mean value of 0.049 mSv/y^1^that was estimated to be the effective dose due to soil radioactivity in Ibadan.[14] Therefore, the risk from radon is found to be higher than that from (outdoor) terrestial radiation.

Nigeria, in common with other African countries, has not yet formulated national directives to enforce radon limits in dwellings and workplaces. There is no general awareness or factual knowledge about radon and its health hazards. Radon concentrations were found to be moderately high in some of the offices surveyed due to the poor ventilation system and the covering used in the offices, e.g., carpet, linen, etc..

### Effects of air-conditioners

All the offices, except one, were well air-conditioned by independent type air conditioners throughout the working hours. In the only office without an air-conditioner, the ^222^Rn value was found to be 346 Bq/m^3^, while the values for the remaining offices ranged from 157–495 Bq/m^3^with a mean of 291 ± 58 Bq/m^3^. It is a common practice in Nigeria for air-conditioned offices to have all their windows closed all the time, even after office hours when the air-conditioning is switched off. It is widely agreed that the principal source of ^222^Rn in houses is the soil gas in the surroundings, but it could be reduced by a high ventilation rate. Adequate supply of outside air, typically delivered through the air-conditioning (HVAC) system, is necessary in any office environment to dilute indoor radon concentrations because less ventilation allows radon to build up.[15]

### Effects of covering material on indoor ^222^Rn concentration

Most of the offices (62.5%) had paint as the covering material for the walls and ceilings and carpets for the floors. The second most common combination (29.2%) was paint for the walls and ceilings, and ceramic tile for the floors. In the remaining offices (8.3%) paint was used as the covering material for walls and ceilings, with linen used as the covering for the floors. These are hereafter referred to as combinations (A), (B), and (C). The radon concentrations for offices with different covering materials for walls, ceilings, and floors are presented in Table 2. The average radon concentrations in the offices with combinations (A) and (C) were about the same, while the radon concentration for combination (B) was a little higher than that for (A) and (B). A person working in an office with combination (B) is estimated to receive an average annual effective dose higher (about 0.04 mSv more) than one working in an office with combinations (A) and (C).

**Table 2:** The radon concentration (A_Rn_) and annual effective dose for offices with different covering materials for walls, ceilings, and floors

*Combination*	*Wall*	*Ceiling*	*Floor*	*Sites (%)*	*A_Rn_(Bq /m^3^)*	*Annual effective dose (mSv /y)*
(A)	Paint	Paint	Carpet	62.5	287.9	1.82
(B)	Paint	Paint	Ceramic tiles	29.2	294.5	1.86
(C)	Paint	Paint	Linen	8.3	288.0	1.82

## Conclusions

A survey of ^222^Rn measured by passive type radon monitors (CR-39) in offices in Nigeria oldest university campus was conducted over a period of 3 months. Based on 24 measurements, the arithmetic mean and standard deviation of the ^222^Rn concentrations were 293.3 and 79.6 Bq/ m^3^, respectively. The geometric mean was 283.6 Bq/ m^3^. The values are below the upper value of the ICRP reference level of 1500 Bq/m^3^[2] for workplaces. In Nigeria’s oldest university, the mean annual effective dose to the public from ^222^Rn was calculated to be 1.85 mSv/ y. The commonest combination of covering materials for the walls, ceilings, and floors in Nigeria’s oldest university were paint, paint, and carpet (A); paint, paint, and ceramic tile (B), and paint, paint, and linen (C), respectively. The average radon concentrations in offices with combinations (A) and (C) were about the same, while that for offices with combination (B) was a little higher than those with (A) and (B). A person working in an office with combination (B) can receive an average annual effective dose that is higher (by a small amount 0.04 mSv) than one working in an office with combinations (A) and (C)
